# Operando Decoding of Surface Strain in Anode‐Free Lithium Metal Batteries via Optical Fiber Sensor

**DOI:** 10.1002/advs.202203247

**Published:** 2022-07-21

**Authors:** Yanpeng Li, Yi Zhang, Zhen Li, Zhijun Yan, Xiangpeng Xiao, Xueting Liu, Jie Chen, Yue Shen, Qizhen Sun, Yunhui Huang

**Affiliations:** ^1^ School of Optical and Electronic Information National Engineering Research Center of Next Generation Internet Access‐system Wuhan National Laboratory for Optoelectronics Huazhong University of Science and Technology Wuhan Hubei 430074 China; ^2^ State Key Laboratory of Material Processing and Die & Mould Technology School of Materials Science and Engineering Huazhong University of Science and Technology Wuhan Hubei 430074 China

**Keywords:** anode‐free lithium metal batteries, fiber Bragg grating, in situ strain monitor

## Abstract

With zero excess lithium, anode‐free lithium metal batteries (AFLMBs) can deliver much higher energy density than that of traditional lithium metal batteries. However, AFLMBs are prone to suffer from rapid capacity loss and short life. Monitoring and analyzing the capacity decay of AFLMBs are of great importance for their future applications. It is known that the capacity fade mainly comes from the formation of solid electrolyte interphase species and dead lithium, which leads to irreversible volume expansion. Therefore, monitoring and distinguishing the irreversible volume expansion or reversible volume expansion are the key points to analyze the capacity fade of AFLMBs. Herein, an applicable technique based on optical fiber sensors to characterize and quantize the volume change of AFLMBs is developed. By attaching fiber Bragg grating (FBG) sensors onto the surface of the multilayered anode‐free pouch cells, the strain evolution of the cells is successfully monitored and correlated with their electrochemical properties. It is found that the decline of surface strain fluctuation amplitude caused by the loss of active lithium is the leading indicator of battery failure. The proposed sensing technique has excellent multiplexing capability that can be considered as an elementary unit for capacity fade analysis in next‐generation battery management system.

## Graphical abstract

Monitoring and analyzing the capacity decay of anode‐free lithium metal batteries are of great importance for their future applications. The strain evolution contains abundant information about capacity decay, which is collected and decoded via optical fiber sensor. The small size and excellent multiplexing capacity of optical fiber sensor make it a powerful tool for capacity fade analysis in practice.

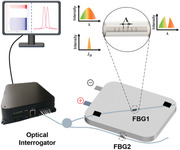

## 1 Introduction

High‐energy‐density rechargeable batteries are indispensable in the applications of consumer electronics, electric vehicles, and energy storage.^[^ ^1^ ^]^ AFLMBs that employ a fully lithiated cathode and a bare current collector can deliver the maximum possible energy density from any given cathode system.^[^ ^2^ ^]^ Therefore, they are regarded as the most promising candidate for next‐generation rechargeable batteries with high energy density. However, AFLMBs also suffer from severe capacity fade since there is no excess lithium to replenish the loss of active lithium due to the formation of electrically isolated lithium (called “dead” lithium) and solid electrolyte interphase (SEI).^[^ ^3^ ^]^ Up to now, many strategies have been proposed to extend the cycle life of AFLMBs, including electrolyte optimization,^[^ ^4^ ^]^ current collector modification,^[^ ^5^ ^]^ and the construction of all‐solid‐state AFLMBs.^[^ ^2^, ^6^ ^]^ For understanding the electrochemical mechanism and further improving the performance of AFLMBs, monitoring the health evolution and analyzing the capacity degradation of battery are very important and even challenging.^[^ ^7^ ^]^

The capacity fade is generally related with the loss of active lithium, which mainly comes from the formation of SEI species and dead Li.^[^ ^8^ ^]^ These two inactive Li species will lead to irreversible volume expansion.^[^ ^9^ ^]^ It is known that the active Li species also undergo volume changes during intercalation and deintercalation of lithium, and hence result in volume expansion periodically in the batteries.^[^ ^10^ ^]^ The reversible volume expansion is closely related with the releasable capacity.^[^ ^11^ ^]^ Therefore, finding a way to monitor and distinguish the reversible and irreversible volume expansion is crucial to characterize the capacity fade of AFLMBs. At present, the common parameters used to evaluate the influence of volume expansion are thickness,^[^ ^12^ ^]^ strain,^[^ ^13^ ^]^ or stack stress.^[^ ^11^, ^14^ ^]^ In 2017, Dahn et al.^[^ ^12^ ^]^ reported the volume, pressure, and thickness evolution of Li‐ion pouch cells with silicon‐composite negative electrodes by using an electrical force sensor and a customized fixture. With similar devices, Koerver et al.^[^ ^14c^ ^]^ measured stress evolution for several different combinations of cathode and anode materials during cycling, and found that the huge difference in partial molar volume between anode and cathode materials is the key factor resulting in stress evolution. McDowell et al.^[^ ^14a^ ^]^ investigated the stress evolution in solid‐state batteries and demonstrated that different alloy anode materials (Sb, Sn and Si) differed in the chemo‐mechanical effect and the size of the anode materials was also a critical factor. Limited by the relatively complex structure and poor multiplexing capability of electrical force sensors, the proposed devices are mainly used to research the internal electrochemical mechanisms of lithium batteries.^[^ ^15^ ^]^ The potential for analyzing the degradation of battery through monitoring volume change has not been excavated. Therefore, a powerful and flexible sensing technology is indispensable to discern the state of batteries behind the volume change.^[^ ^15^, ^16^ ^]^

Optical fiber sensors can measure the strain conveniently, which can be applied to characterize and quantize the volume change of pouch cell.^[^^]^ The competence mapping of the optical fiber sensor is presented in Figure S1, Supporting Information. Compared with the electric force sensors, they have smaller size, longer service life, and stronger electrochemical stability. What is more, the multiplexing capacity can be extended up to 1000 by utilizing the optical power variation of reflected sensing signal in both time domain and wavelength domain.^[^ ^18^ ^]^ The excellent multiplexing capability makes them ideal sensors for in situ characterizing the volume change in the practical battery system that has hundreds of cells.

In this work, an in situ optical fiber sensor derived monitoring technique is developed to realize the strain evolution and its operando decoding of LiNi_0.5_Mn_0.3_Co_0.2_O_2_ (NMC_532_)‐based AFLMBs in full life circle. Multiple characterization methods including scanning electron microscopy (SEM) and ultrasonic imaging are utilized to provide abundant evidence. It is found that the decline of surface strain fluctuation amplitude caused by the loss of active lithium is the leading indicator of complete failure. The proposed sensing technologies can offer a fundamental tool for capacity fade analysis in AFLMBs.

## 2 Results and Discussion

**Figure 1:**
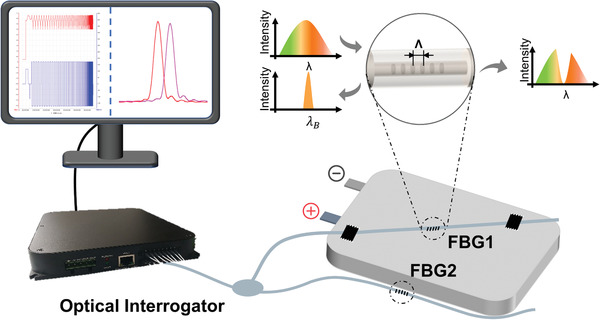
The schematic diagram of experimental setup for strain monitoring of AFLMB.

The optical fiber sensor used in this work is FBG. As shown in the inset of **Figure** **1**, it has a periodic modulation of refractive index along the core of fiber. The resonant wavelength of FBG (also called Bragg wavelength) can be described as:^[^ ^19^ ^]^

(1) $$\lambda_{B}=\;2n_{\mathrm{eff}}\Lambda$$

where *n*_eff_ is the effective refractive index of light transmitting in the fiber and Λ is the grating period of FBG. Owing to the elastic‐optical effect and thermo‐optical effect, the refractive index of optical fiber will change when it is stretched or its temperature varies. As a result, *n*_eff_ will change and leads to the shift of Bragg wavelength.

According to the working principles of FBG, the schematic diagram of experimental test of surface strain is designed and shown in Figure 1 (The picture of real measurement system is shown in Figure S2, Supporting Information). It contains an optical interrogator (Gaussian optics OPM‐T1620 with the wavelength accuracy of 1 pm and sampling frequency of 1 Hz) and two FBGs with different Bragg wavelength. Here, the optical interrogator serves as light source, as well as the data acquisition system. In the experiment, one FBG (FBG1) is fixed on the surface of battery by using adhesive tape to monitor the strain and the other one with different resonant wavelength (FBG2) is freely placed on the side of battery to monitor the ambient temperature. Thus, the wavelength shift of FBG1 and FBG2 can be described as:

(2) $${\Delta \lambda }_{\mathrm{FBG}1}=K_{\varepsilon }\;\varepsilon +K_{T}\Delta T$$

(3) $${\Delta \;\lambda }_{\mathrm{FBG}2}=K_{T}\Delta T$$

where *ε* and *T* are the strain and temperature of FBG, *K*_*ε*_ and *K*_T_ are the coefficient related to the elastic‐optical effect and thermo‐optical effect, respectively. At this moment, the surface strain evolution of battery can be calculated as:

(4) $$\varepsilon \;=\frac{{\Delta \lambda }_{\mathrm{FBG}1}-{\Delta \lambda }_{\mathrm{FBG}2}}{K_{\varepsilon }}\;$$

In this work, the measured wavelength shifts of FBG1 and FBG2 are shown in Figure S3a,b, Supporting Information. The strain rate constant (*K*_*ε*_) response of FBG sensors is 0.854 pm µ*ε* ^−1^ (Figure S4, Supporting Information). According to Equation (4), the surface strain evolution of battery during cycling can be extracted and quantized. The specific processes of measurement are demonstrated in the Experimental Section.

**Figure 2:**
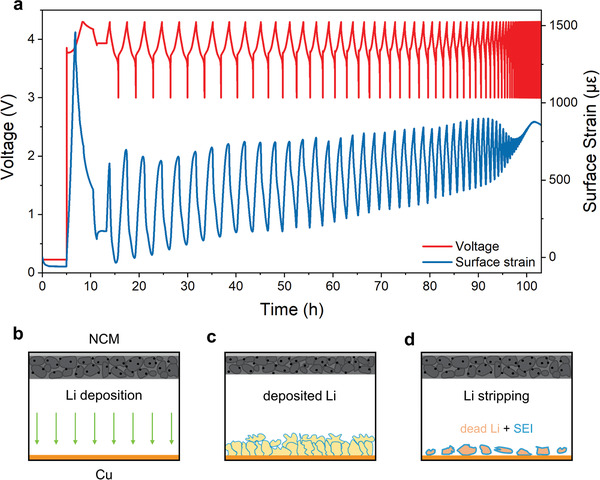
The surface strain evolution of the anode‐free pouch cell during cycling: from the first cycle to complete failure. a) The galvanostatic curves and strain signal of the lifetime of the pouch cell. b‐d) Schematic of Li deposition/stripping on the Cu foil in anode‐free batteries.

The galvanostatic cycling curves and the corresponding surface strain evolution are shown in **Figure** **2**. It is noted that the influence of ambient temperature has been removed through the method mentioned above. It can be found that the surface strain increases and decreases regularly during the charge and discharge process. It is well consistent with the volume change caused by the intercalation/de‐intercalation of lithium during cycling. As illustrated in Figure 2b,c, Li ions are deposited on Cu foil during charge. According to the report of Koerver et al.,^[^ ^14c^ ^]^ all volume change aroused by (de)intercalation of lithium can be attributed to the difference in partial molar volume of lithium within the cathode and anode materials. The partial molar volume of lithium can be defined as:

(5) $${\bar{V}}_{m}\;\left(\mathrm{Li}\right)={\left(\frac{\partial V}{\partial n\left(\mathrm{Li}\right)}\right)}_{T,p,n_{i}\neq n_{\mathrm{Li}}}\;$$

where *V* is the total volume and *n*(Li) is the differential change in the quantity lithium in the material. The partial molar volume of lithium within different materials covers widely. As for lithium metal, it has a partial molar volume of 12.97 cm^3^ mol^−1^ at 25 °C. The NMC_532_ used in this work has a low ${\bar{V}}_{m}\left(\mathrm{Li}\right)$ value which varies between 1 and 5 cm^3^ mol^−1^ depending on the state of charge (SOC). The ${\bar{V}}_{m}\left(\mathrm{Li}\right)$ of lithium is obviously higher than that of NMC_532_. Therefore, the net volume expansion in the anode is larger than the volume contraction in the cathode during charge, which results in a net volume expansion, as demonstrated in Figure 2b,d. To adapt the volume change of battery, the cell skin is stretched, causing the positive surface strain of FBG.^[^ ^13b^ ^]^ In the discharge process, the situation is reversed. It is noted that there is some lithium staying at anode as the SEI and dead Li. Therefore, the total volume becomes larger and larger during cycling, and the initial strain increases at the beginning of each cycle as well.

**Figure 3:**
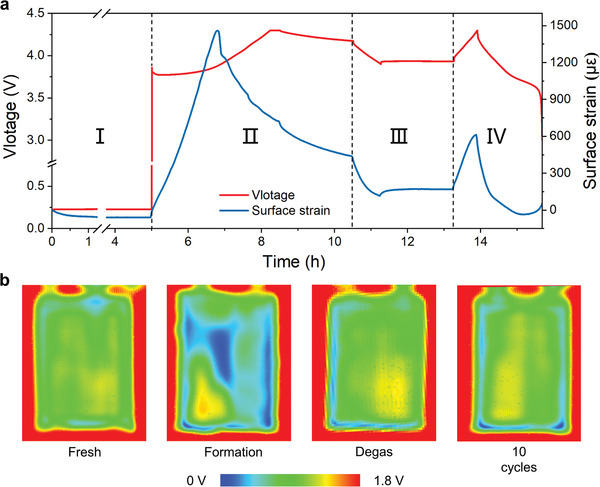
The formation process of the anode‐free pouch cell. a) The galvanostatic curves and strain signal of formation process. The pouch cell was charging at 0.2 C to 4.3 V and charging at 4.3 V to a cutoff current of 0.05 C. Subsequently, the pouch cell was discharging at 0.2 C to 3.9 V. b) Ultrasonic transmission mappings of the pouch cell before cycling, after formation, degassing, and ten cycles.

Fixing the FBG sensors on the surface of the battery, the stretch and contract of cell skin caused by the periodic change and irreversible change in volume can be recorded and quantized in the whole life time. After that, the formation process of AFLMB is first studied in detail. The specific method of formation is introduced in Experimental Section.^[^ ^4^, ^20^ ^]^ For clear presentation, the surface strain evolution and its voltage curve in formation process are replotted in **Figure** **3**a. In Figure 3a, the formation is artificially divided into four regions. In region I, the battery is held at open circuit for five hours to reach a relatively stable state after it is bounded with the FBG sensors. From the strain evolution curve, it can be found that the strain decreases slowly and turns into ≈−64 µ*ε* after 1 h and then becomes constant. The decrease of surface strain is related to the decrease of the initial stack pressure, which may come from the deformation of electrode materials and separators in pouch cell skins.^[^ ^14b^ ^]^ An interesting finding in region II is that the surface strain reaches the maximum at the middle of charge, and the peak value (≈1460 µ*ε*) is much higher than those of other cycles. The charge or discharge capacity is usually maximal at the first cycle and the surface strain is expected to reach the maximum at the end of charge. Actually, the accumulation of gas in the formation also plays an important role in the surface strain evolution.^[^ ^20^, ^21^ ^]^ It is known that a lot of gas is yielded along with the formation of SEI at first cycle. The accumulation of gas leads to the increase of strain. Once the SEI is basically formed, gas production decreases significantly. Meanwhile, part of the gas is expelled into the prepared gas bag. As a result, the accumulation of gas reaches the maximum at the middle of charge. Therefore, the strain increases acutely at first and decreases dramatically due to less gas accumulation.

To investigate the gassing behavior of the pouch cells in detail, ultrasonic imaging technique,^[^ ^21^ ^]^ developed by our group, is also employed. Ultrasonic imaging technique is an effective non‐destructive technique to investigate the state of the electrolyte and gas inside a pouch cell. Through detecting the peak–peak values of ultrasound signal and converting them to color scale, a pseudo‐color image is formed. In an ultrasound image, the red and blue regions, respectively, correspond to the high and low transmission regions, representing different wetting states and gas behavior. According to the working principle of ultrasonic imaging technique, the poor wettability of electrolyte and the production of gas will both cause the low transmission regions (blue regions). Therefore, the battery is imaged first to ensure that the pouch cell was completely wetted, as shown in Figure 3b. After formation, the blue regions appear, which indicates that the gas accumulates dramatically during formation, corresponding to the sharp rise of strain as well. After degassing over ten cycles, almost no gas is produced in the pouch cell. Hence, the significant deviation of strain peak does not appear in the subsequent cycles.

In region III (Figure 3a), the signal of strain increases slowly in the static stage after discharge, similar to the increasing voltage caused by polarization. The volume change caused by ion diffusion in graphite anode has been reported by Raghavan et al.^[^ ^13a^ ^]^ The corresponding signal can be ascribed to lithium‐ion diffusion in cathode material, which deserves further study in future.

**Figure 4:**
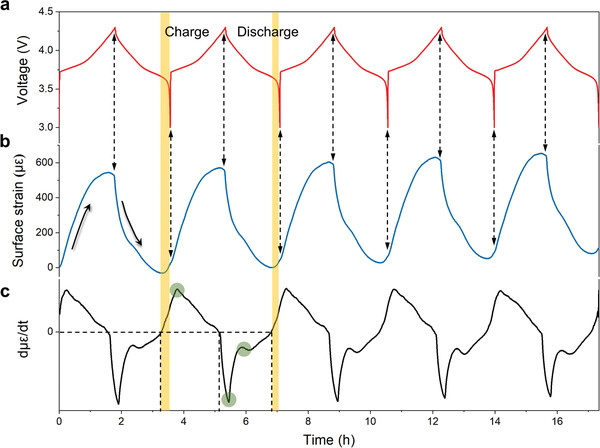
Electrochemical cycling and strain signal monitored by fiber Bragg grating sensors of anode‐free pouch cells. a) Galvanostatic cycling of anode‐free pouch cell with NMC_532_ cathodes. b,c) The strain signal and the derivative of the strain with time.

To further analyze the relationship between voltage and surface strain, we extract a portion of the plot to show how the strain changes with cycling. In **Figure** **4**a,b, it is found that the strain signal swings with voltage. This means that the surface strain is closely related to the SOC, providing a new strategy to monitor SOC of batteries. It is noted that the strain reaches the lowest and increases subsequently before the end of discharge. This tendency happens over five cycles, which can be attributed to the properties of anode‐free cell. For the anode‐free batteries, the thick SEI formatted in the formation process, exhibiting a porous and rigid structure. At the end of discharge, the rigid and porous SEI does not contract with the stripping of lithium. Instead, the NMC_532_ material expands with intercalation of lithium, causing the increase of strain signal. These findings are well consistent with the difference of partial molar volume.

Furthermore, the slopes of strain changes during charge and discharge also provide information to reveal the internal electrochemical mechanisms. Figure 4c shows the time derivate of the strain (dµ*ε*/dt) over five cycles. A primary phenomenon attained from the curves is that, the rate of change for the strain is not constant during charge and discharge, and both present a downward trend. The slope of strain curve is greater near the beginning of charge and discharge, and tends to zero at the end. The decrease of slope is probably attributed to the variational ${\bar{V}}_{m}\left(\mathrm{Li}\right)$ within NMC_532_ depending on Li content. For instance, ${\bar{V}}_{m}\left(\mathrm{Li}\right)$ within NMC_532_ increases with decreasing Li content, resulting in an increased rate of contraction in cathode material. Considering that SEI is relatively stable after breaking and repairing at the beginning, its porosity and rigidity endow it with high resistance to expand. While, ${\bar{V}}_{m}\left(\mathrm{Li}\right)$ in Li metal is approximately constant. As a result, the slope of the strain curves decreases gradually.

Interestingly, the peaks and valley marked by the green circles in dµ*ε*/dt curves obviously show similar variations over five cycles, however, they represent different rate of volume change. This can be explained by the structural change in active materials during charge and discharge, which indicates that the current technique is potential to monitor the phase transition of the active materials.

The reversible and irreversible volume expansions are closely related to the capacity fade of batteries, and the surface strain is effectively used to characterize the influence of volume expansion. The maximum and minimum of strain evolution in each cycle are extracted and presented in Figure S5, Supporting Information. It can be found that the minimum, representing the cumulative volume caused by SEI and dead lithium, tends to increase after each cycle. It can be demonstrated by the fact that the endless reaction between lithium and electrolyte not only yields dead lithium, but also makes the SEI thicker, resulting in the loss of active lithium and irreversible volume expansion of pouch cell.

**Figure 5:**
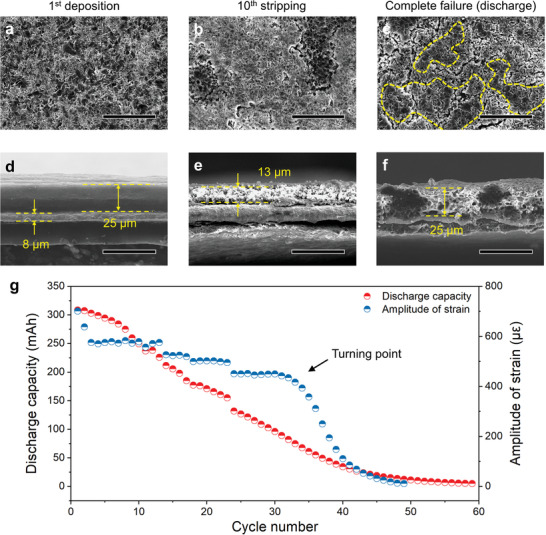
a) The amplitude of strain signal and capacity vary with the cycle number. Morphology of anode before and after cycling. b,d) Top surface and e–g) cross‐section images of the first deposition, tenth stripping, and complete failure. Scale bar, 50 µm.

To prove this point, the morphological changes of electrodes were analyzed. The electrodes were cut into pieces, respectively, after the first deposition, tenth stripping, and complete failure, and then characterized. The pristine current collector was bare Cu foil with thin thickness of 8 µm. **Figure** **5**a–f shows the top‐view and cross‐section SEM images of the anode. After the first deposition, lithium metal was deposited on Cu foil densely (≈25 µm) (Figure 5a,d). In the subsequent cycle, the SEI and dead Li formed and accumulated in current collector (Figure 5b,c,e,f). Additionally, the anode became thicker with cycling and its top‐view became rougher after complete failure. Meanwhile, the thickness of the whole pouch cell was measured by micrometer caliper. As shown in Figure S6, Supporting Information, with the accumulation of SEI and dead Li, the whole pouch cell become thicker. As a result, the skin of pouch cell was stretched. This phenomenon is consistent with the increase of the minimum strain in each cycle.

To reveal the relationship between surface strain change and capacity fade directly, the amplitude of strain evolution and the capacity of pouch cell are plotted in Figure 5g. It can be seen that the capacity of the pouch cell tends to 0 after about 40 cycles (≈80 h) and the amplitude step reduces during cycling. Similarly, the amplitude of surface strain decreases during cycling as well. It is worth noting that there is a turning point at about 30th cycle which is ten cycles earlier than that of complete decay of battery. In order to prove that the observed phenomenon is not an accident, the strain evolution of other cells are monitored and one of them is presented in the Figure S7, Supporting Information. It can be easily found that the amplitude fluctuates in a tight range and after about 30 cycles, the amplitude decreases rapidly. The cycle numbers where turning point appears are summarized in Table S1, Supporting Information. It is obvious that the turning points appear earlier than that of complete decay of battery and the advance cycles are 10, 9, and 11, respectively. The reproducible results indicate that the turning point of strain amplitude can be considered as a leading indicator of complete decay of battery, which makes it possible for monitoring the state of health in real time.

## 3 Conclusion

An in situ technique based on FBG sensor has been developed to monitor the strain evolution during cycling for rechargeable batteries. The operando decoding of strain evolution is realized with the help of multiple characterization methods including SEM and ultrasonic imaging. Taking the anode‐free lithium battery matching NMC_532_ cathode as an example, we find that the strain signal is closely related to SOC and its fluctuation amplitude can be associated with the delivered capacity. Most importantly, the proposed sensing technique can be also used in monitoring the traditional Li‐ion batteries. The small size, low cost, and strong multiplexing capability of the FBG sensors make it possible to be filled in every tiny crevice of packs and modules to monitor each cell in a pack, which offers a unique direct view to understand the internal health and the electrochemical changes of rechargeable batteries and hence enables developing new battery management system.

## 4 Experimental Section

### Electrochemical Testing

The pouch cells without electrolyte were purchased from Nanjing TONGNING New Material Research Institute. The negative electrode was a bare copper foil and the positive electrode was LiNi_0.5_Mn_0.3_Co_0.2_O_2_ (NMC532) material with the loading of 35.6 mg cm^−2^ (two sides). After drying for 12 h at 60 °C, the cells were filled with 650 µL electrolyte in an argon‐filled glove box (H_2_O < 0.1 ppm, O_2_ < 0.1 ppm) and then were vacuum sealed. The electrolyte was purchased from Duoduo Chem Co., Ltd., containing 1.0 m LiTFSI in EC and DEC in a volume ratio of 1:2 v/v with 5% FEC. After filling with electrolyte and resting for 12 h, the pouch cell was charged and discharged initially. At first, the pouch cell was charged at 0.2 C to 4.3 V, then turned into the constant voltage charging mode with a cut‐off current of 0.05 C. Subsequently, the pouch cell was discharged at 0.2 C to 3.9 V. In the charging process of formation, constant current charging and constant voltage charging (CC + CV) were used. The adoption of CV eliminated polarization and obtained higher charge capacity, making most of lithium deposit on the Cu foil.^[^ ^1^ ^]^ Meanwhile, the CV also was designed to take advantage of spending more time in the low anode overpotential region where the SEI was formed via electrolyte solvent and salt decomposition. Subsequently, 3.9 V as cutoff voltage in the discharge was applied. Since the SEI mainly formed in the low anode overpotential region, discharging to lower voltage was of little significance to the formation of SEI. Besides, the lithium left on the Cu foil also improved the conditions for the next lithium deposition. Cycling performances of the pouch cells were measured on a Neware multichannel battery cycler at 0.3 C between 3.0 and 4.3 V. All measurements were tested at 25 °C.

### Strain Measurement

Two FBG sensors were employed to measure the strain for one pouch cell. The first FBG sensor was bonded at the surface of the pouch cell using adhesive, and the other FBG was loosely placed on the side of the pouch cell, considered as reference sensor to compensate temperature variations. Further, the pouch cell was clamped between two stainless steel sheets to obtain an initial stack pressure. The picture of real measurement system is presented in Figure S2, Supporting Information.

### Strain Sensitivity Calibration

The strain sensitivity measurements were performed using translation stages (Newport, M‐ILS300LM‐S) and both ends of FBG sensor were fixed onto the stages using the fiber holder. The distance between the fixed points is 200 mm. One stage remained static, whereas the other moved by a step of 0.1 mm (500 µ*ε*). The strain was maintained for 60 s at each level, and the tests were performed in the 0–4500 µ*ε* range.

### Materials Characterization

The morphologies of electrodes were characterized by a field‐emission SEM (FEI Nova NanoSEM450). All samples were washed two times with DEC and dried naturally for 5 min. The above operations were finished in an argon‐filled glove box (H_2_O < 0.1 ppm, O_2_ < 0.1 ppm).

### Ultrasonic Imaging

An ultrasonic battery scanner, purchased from Jiangsu Jitri‐Hust Intelligent Equipment Technology Co., Ltd., was employed to get ultrasonic transmission mappings. The pouch cell was fully immersed in the water‐filled test tank, and then a pair of ultrasonic focusing transducers (2 MHz frequency) was placed on each side. The pouch cell was penetrated by ultrasound waves from a transducer on one side and the transmitted waves were received by another transducer. Subsequently, through self‐defined mapping algorithm, the peak‐to‐peak values of the transmitted waveforms were converted to a color scale to get a colorful ultrasonic imaging.

## Conflict of Interest

The authors declare no conflict of interest.

## Supporting information

Supporting Information

## Data Availability

The data that support the findings of this study are available from the corresponding author upon reasonable request.
