# Supplementary material for: Operando Decoding of Surface Strain in Anode‐Free Lithium Metal Batteries via Optical Fiber Sensor
Source: Adv Sci (Weinh). 2022 Jul 21;9(26):2203247. doi: 10.1002/advs.202203247 (PMC9475526; doi:10.1002/advs.202203247)
Supplement: Supplementary file 1 — Supporting Information [file ADVS-9-2203247-s001.pdf]

ADVANCED SCIENCE
Open Access

Supporting Information

for *Adv. Sci.*, DOI 10.1002/advs.202203247

Operando Decoding of Surface Strain in Anode-Free Lithium Metal Batteries via Optical Fiber Sensor

Yanpeng Li, Yi Zhang, Zhen Li, Zhijun Yan, Xiangpeng Xiao, Xueting Liu, Jie Chen, Yue Shen, Qizhen Sun* and Yunhui Huang**

Supporting Information

Early warning of capacity fade in anode-free lithium metal batteries via optical fiber sensor

Yanpeng Li[+], Yi Zhang[+], Zhen Li, Zhijun Yan, Xiangpeng Xiao, Xueting Liu, Jie Chen, Yue Shen, Qizhen Sun*, Yunhui Huang**

Supplementary Information

The comparison between optical fiber sensors and traditional electronic sensor

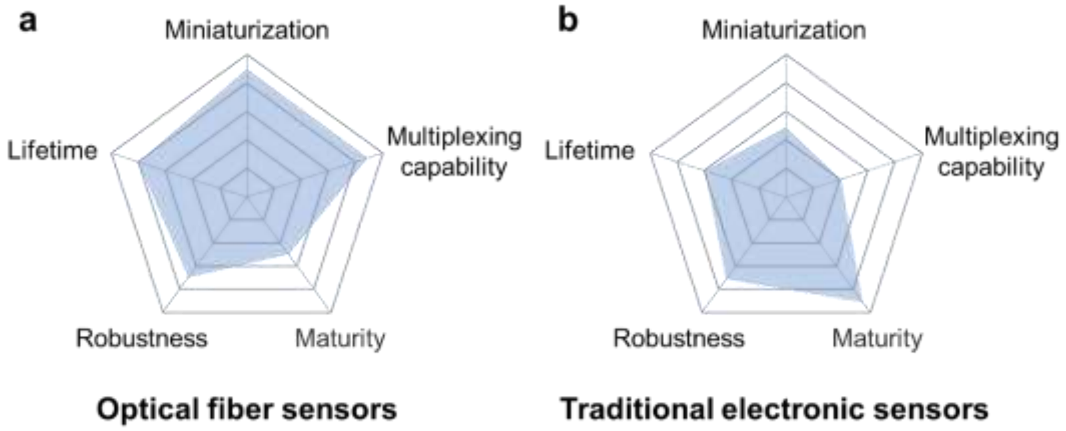

Supplementary Fig S1. The competence mapping of optical fiber sensors and traditional electronic sensors.

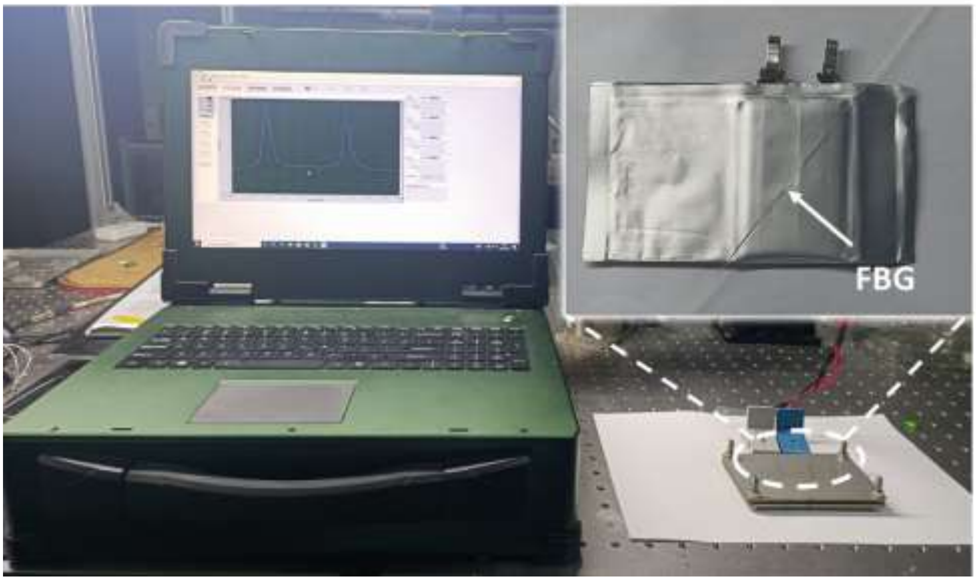

Supplementary Figure S2. The picture of real measurement system.

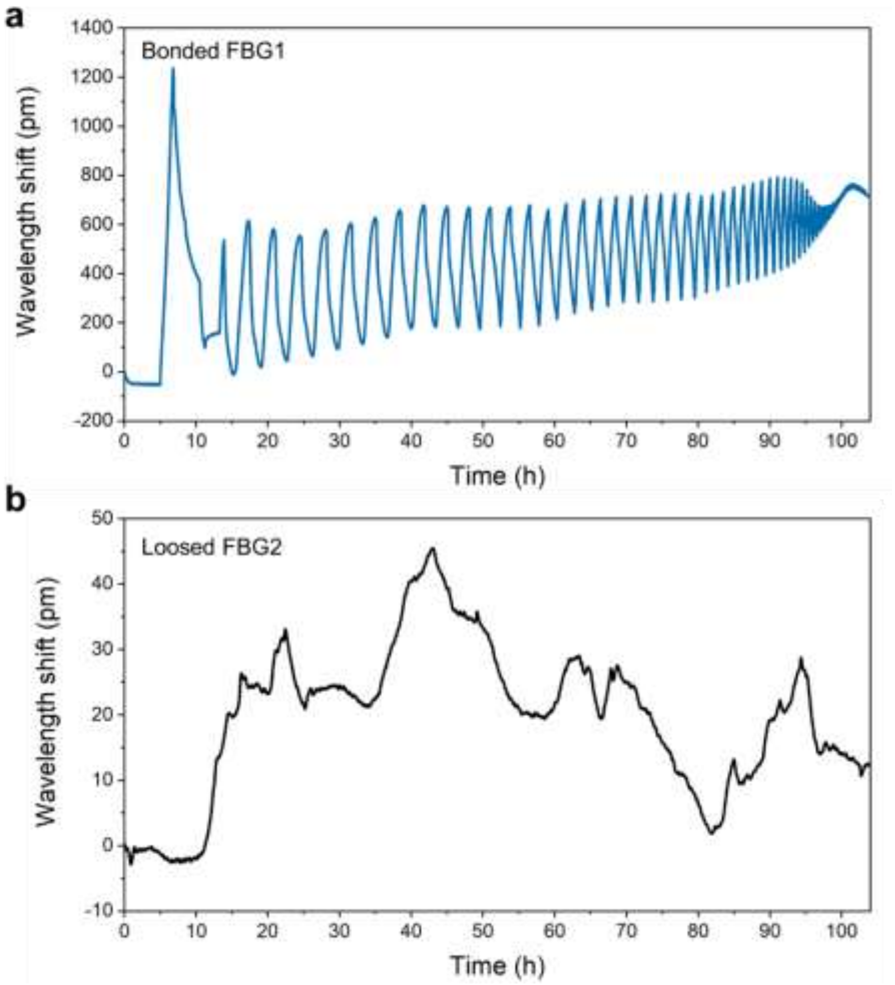

Supplementary Fig S3. The original wavelength shifts of FB1 and FBG2.

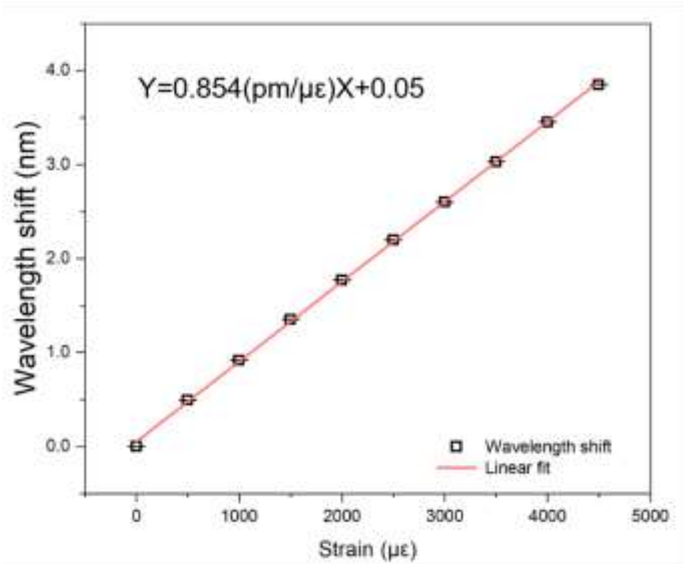

Supplementary Figure S4. The wavelength responses of FBG to strain. The points are linearly fitted by the red line.

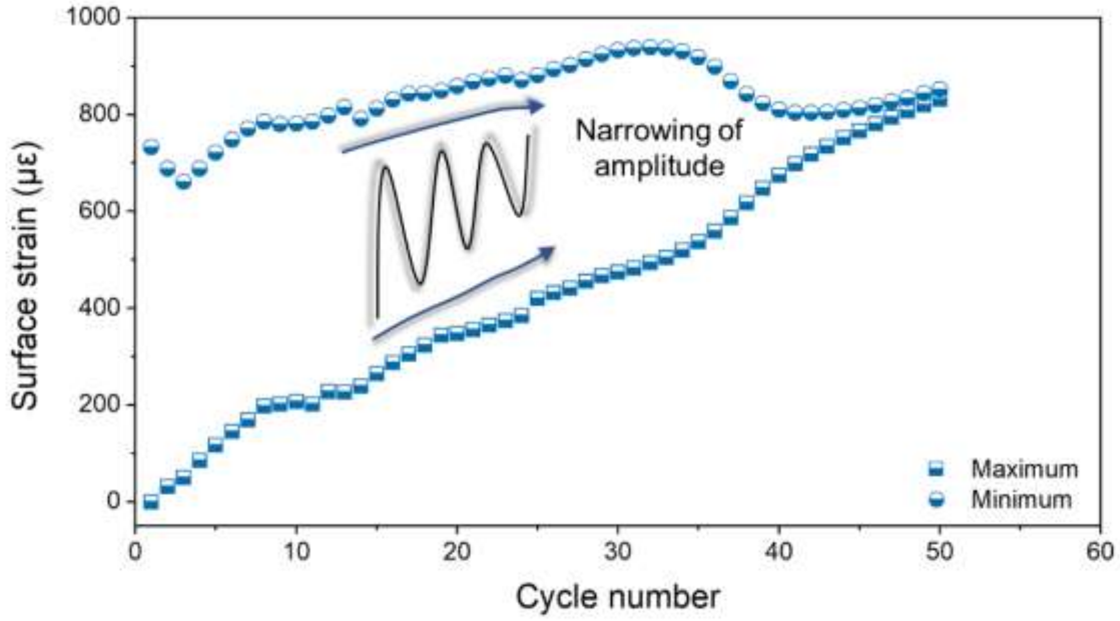

Supplementary Figure S5. The maximum and minimum strain signal of pouch cell in every cycle.

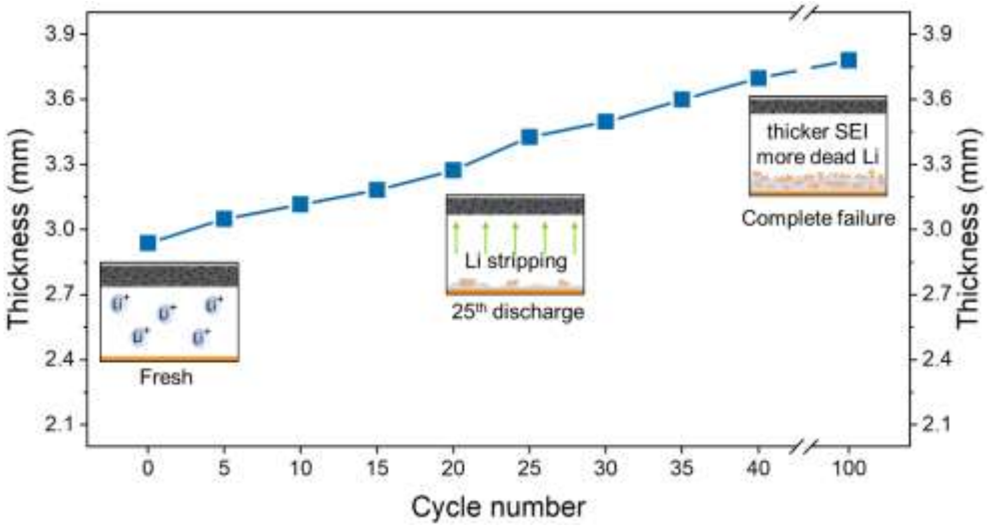

Supplementary Figure S6. The thickness of the whole pouch cell, measured by micrometer caliper. The thickness is the average value of 20 measuring points on the pouch cell. The inset represents schematic of the thickening of the SEI and dead Li in Cu foil.

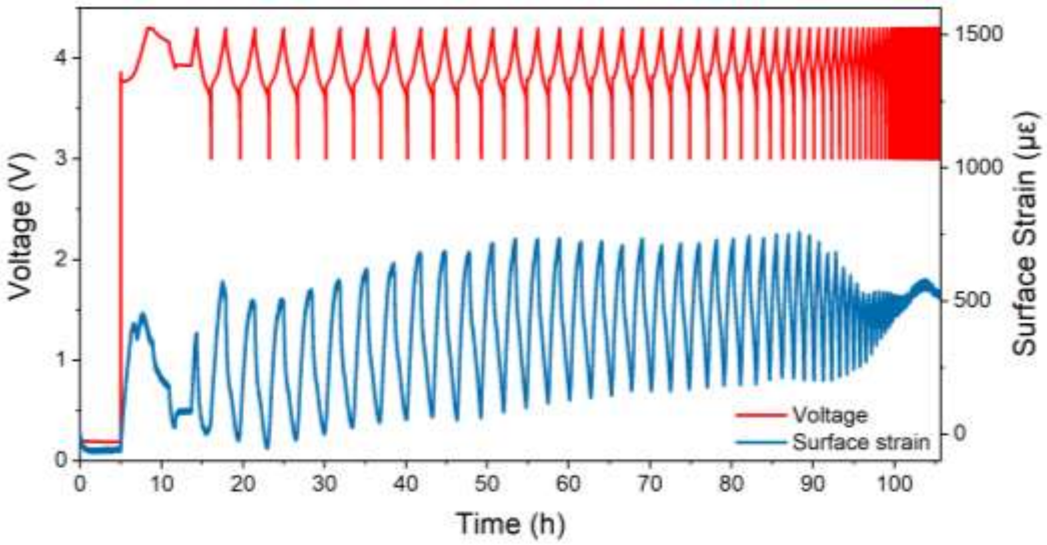

Supplementary Figure S7 |The strain evolution of another cell, the turning point of amplitude appear at 30th cycle and the completely failure of battery at 40th cycle

**Times**	**1**	**2**	**3**
**Cycle number that turning point appears**	**30**	**31**	**30**
**Cycle number that complete failure appears**	**40**	**40**	**41**

Supplementary Table S1. The summary of cycle number that turning point appear in another three measurements.
